# How to Scan Patients With Severe Aortic Stenosis

**DOI:** 10.1016/j.jaccas.2025.104415

**Published:** 2025-07-30

**Authors:** Angelica Poniros, Kerry Nevin, Gilbert H.L. Tang, Lucy M. Safi

**Affiliations:** a Division of Cardiology, Westchester Medical Center, Valhalla, New York, USA; b Division of Cardiology, Mount Sinai Fuster Heart Hospital, Icahn School of Medicine at Mount Sinai, New York, New York, USA; c Department of Cardiovascular Surgery, Mount Sinai Health System, Mount Sinai Heart Fuster Hospital, Icahn School of Medicine at Mount Sinai, New York, New York, USA

**Keywords:** aortic valve, Doppler ultrasound, echocardiography, imaging, stenosis

## Abstract

**Objectives:**

Aortic stenosis (AS) is a common yet critical valvular pathology that requires precise diagnosis and severity assessment. This paper aims to provide a step-by-step echocardiographic guide for sonographers, trainees, and allied professionals to help with image acquisition and diagnosis.

**Key Steps:**

Assessment of AS involves obtaining high-quality images in multiple windows and various probes.

**Potential Pitfalls:**

Key challenges include measurement errors, misinterpretation of Doppler signals, improper alignment of the Doppler cursor, and poor-quality acoustic windows due to patient body habitus.

**Take-Home Message:**

A systematic approach to image acquisition with understanding of common pitfalls is essential for the imager and accurate diagnosis and management of AS.

Aortic stenosis (AS) is one of the most prevalent valvular diseases and particularly affects the aging population. Screening and appropriate identification of AS is important because patients may remain asymptomatic until late in the disease process. Accurate and timely diagnosis is critical to optimizing patient outcomes because disease progression can lead to cardiac failure, arrhythmia, or death. Usually AS is identified by murmur on examination, and transthoracic echocardiography is the gold standard for diagnosis.^1^ This paper presents a comprehensive how to guide for the echocardiographic identification and diagnosis of AS. This guide is designed to enhance the skillset of sonographers, physician trainees, and other allied health care professionals, providing a systemic approach including key imaging views, measurements, and hemodynamic assessments necessary for diagnosis.

**Take-Home Messages**

• Aortic stenosis is a critical valvular disease that requires precise echocardiographic assessment for accurate diagnosis and management.
• A step-by-step approach to transthoracic imaging, including proper image acquisition, Doppler alignment, and hemodynamic assessment, is essential to avoid common pitfalls and to ensure reliable measurements.
• Recognizing challenges such as patient body habitus, spectral Doppler misalignment, and incorrect sample volume placement is crucial for optimizing diagnostic accuracy.
• When performed correctly, echocardiography serves as a powerful, cost-effective tool for early detection, leaflet to timely diagnosis, and intervention.

## Transthoracic Imaging Procedural Steps (Figure 1)

1. Using your transthoracic echocardiogram imaging transducer, start your protocol in parasternal long axis (PLAX) to assess left ventricular ejection fraction, left ventricular wall thickness, and the aortic valve (AV) and mitral valve (Figure 2, Video 1).
2. Zoom on the AV and mitral valve in PLAX and evaluate with and without color Doppler (Figure 3, Video 2).
3. PLAX: measure left ventricular outflow tract (LVOT) diameter (in mid-systole), sinus of Valsalva diameter, aortic annulus diameter, ST-segment junction diameter, and ascending aorta diameter (Figure 4).
4. Parasternal short axis: interrogate AV morphology (eg, bicuspid, tricuspid valve) and note the location of calcium or presence of raphe (Figure 5, Video 3).
5. Parasternal short axis: perform planimetry of the AV in systole at the leaflet tips. Using biplane or 3-dimensional imaging can help ensure that you are at the leaflet tips when obtaining a direct planimetry. If not at the leaflet tips, the aortic valve area by direct planimetry may be overestimated or underestimated (Figure 6).
6. Apical 4-chamber: evaluate left ventricular ejection fraction (2-dimensional and/or 3-dimensional) and global longitudinal left ventricular strain. Note that apical 4-chamber, apical 2-chamber, and apical 3-chamber views are all required to determine left ventricular global longitudinal strain (Figure 7, Video 4).
7. Apical 5-chamber: color Doppler on the AV reveals a stenotic mosaic color flow pattern across the AV caused by an outflow obstruction and can also show aortic regurgitation. Evaluate the origin of the turbulence to ensure that it originates at the level of the valve and not subvalvular (which can be seen with a subaortic membrane or with LVOT obstruction from hypertrophic cardiomyopathy) or supravalvular (Figure 8, Video 5).
8. Apical 5-chamber: align continuous-wave (CW) Doppler through the AV to obtain AV peak velocity, gradients, and Doppler assessment of aortic regurgitation if present. Measure the peak AV velocity waveform (Figure 9).
9. Apical 5-chamber: align pulse wave (PW) sample volume in the LVOT avoiding the area of flow convergence (Figure 10). Place the PW sample volume in the AV and slowly move the sample volume into the LVOT until you have no aliasing and a clear central Doppler waveform. Placing the sample volume too close to the AV leaflets will overestimate the velocity; conversely, placing the sample volume too ventricular may underestimate the velocity. When measuring the PW velocity, measure the modal velocity (the bright white), which represents the velocity of most of the blood cells.
10. Apical 3-chamber: placing color Doppler on the AV evaluates for the stenotic mosaic color flow pattern caused by an outflow obstruction and also evaluates for aortic regurgitation (Figure 11, Video 6). Like the 5-chamber view, other causes of LVOT obstruction should be excluded.
11. Apical 3-chamber: align CW Doppler through the AV to obtain AV peak velocity, gradients, and Doppler assessment of AR (Figure 12). Measure the peak aortic velocity waveform in the apical view.
12. Apical 3-chamber: align PW sample volume in the LVOT avoiding the area of flow convergence (Figure 13). A similar technique as described for the 5-chamber view may be used to obtain the PW Doppler.
13. Pedoff (nonimaging transducer): obtain gradients from the apical 5-chamber, suprasternal notch, and right parasternal (RPS) positions (Figure 14). The small size of this transducer allows for manipulation between the ribs to best align with the Doppler waveform. Having the volume on when scanning can give auditory feedback to help identify the location of the AV jet. When scanning the RPS view, rotating the patient on their right side (with right arm above head) can best align the ultrasound beam with the trajectory of the aorta. Measure the peak AV velocity of each waveform at all possible imaging acoustic windows. The Doppler signal will be below the baseline when measuring the peak aortic velocity waveform in the apical view. In the suprasternal notch and RPS views, the Doppler waveform will be above the baseline.
14. Calculate the AV area using the continuity equation (Figure 15): use the LVOT diameter obtained from PLAX (step 3), LVOT velocities or velocity time integrals (steps 9 and/or 12), and AV velocities or velocity time integrals (steps 8, 11, and 13). Average 3 values in sinus rhythm and 5 values in atrial fibrillation. It is imperative to measure the AV peak velocity from all possible acoustic windows.

## Potential Pitfalls

Sonographers may run into challenges when evaluating for AS. Some of the common imaging challenges include suboptimal imaging windows due to body habitus, breast implants, or suboptimal Doppler angle alignments. Other pitfalls include mistaking AS velocities for other disease states (eg, obstruction from hypertrophic cardiomyopathy, mitral regurgitation [MR], subaortic membrane). Some helpful hints to remember when scanning are subsequently described.

1. Do not mistake MR velocity for AS velocity. Noting the start time of the Doppler waveform is helpful in differentiating these 2 processes. The MR Doppler waveform is a round holosystolic Doppler that occurs during both the isovolumic contraction time and isovolemic relaxation time periods. Conversely, AS tends to have a rounded but sharper Doppler waveform that occurs in systole after isovolemic contraction time but before isovolemic relaxation time (Figure 16). While scanning, the AV is anterior positioned (toward the front of chest) and the mitral valve is more midline (toward the sternum).

**Figure 1:**
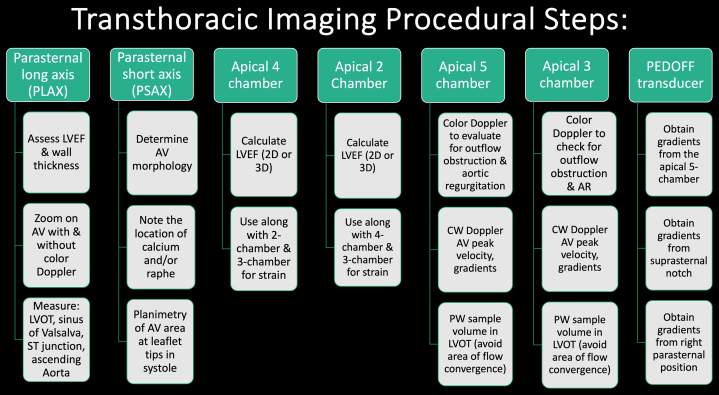
Transthoracic Echocardiogram Procedural Steps for Scanning a Patient with Aortic Stenosis Representative figure illustrating step-by-step transthoracic procedural imaging for evaluation of aortic stenosis. 2D = 2-dimensional; 3D = 3-dimensional; AR = aortic regurgitation; AV = aortic valve; CW = continuous wave; LVEF = left ventricular ejection fraction; LVOT = left ventricular outflow tract; PLAX = parasternal long axis; PSAX = parasternal short axis; PW = pulse wave.

**Figure 2:**
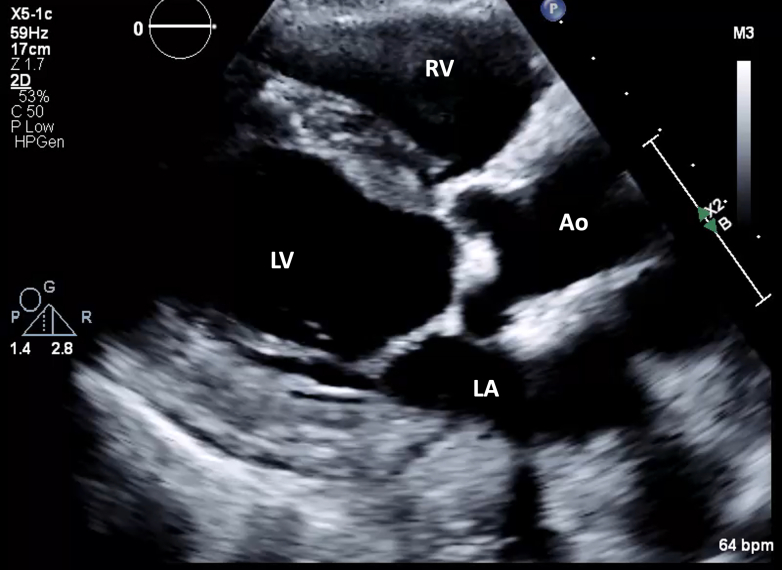
Parasternal Long-Axis View Using your transthoracic echocardiogram transducer, start in the parasternal long-axis view to assess LV ejection fraction and wall thickness. Ao = aorta; LA = left atrium; LV = left ventricular; RV = right ventricular.

**Figure 3:**
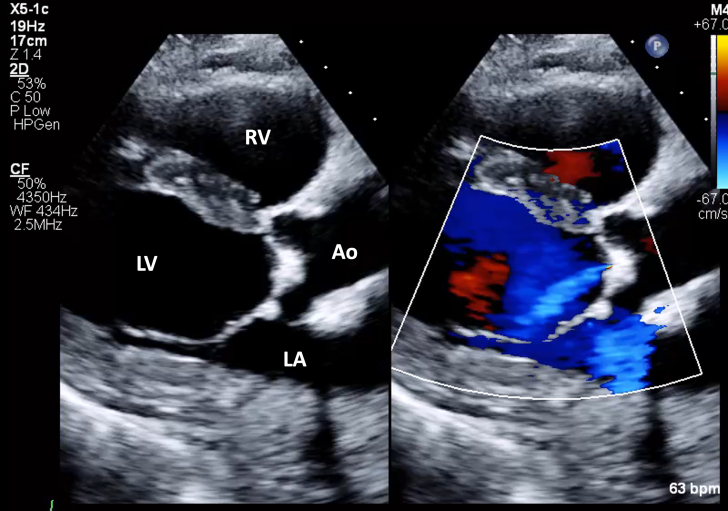
Zoomed Parasternal Long-Axis View With and Without Color Doppler Zoomed in image of the aortic valve in parasternal long-axis view with and without color Doppler. Abbreviations as in Figure 2.

**Figure 4:**
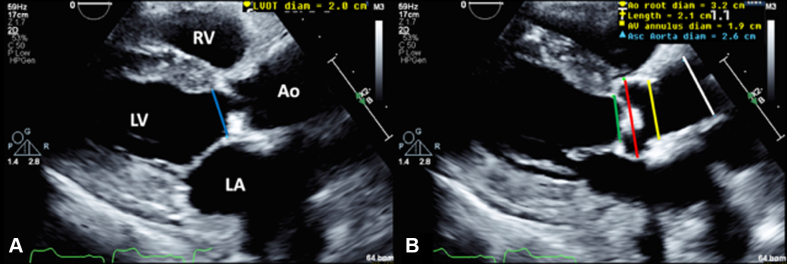
LVOT and Aortic Root Measurements Parasternal long-axis view measurements of the left ventricular outflow tract diameter in mid-systole (left, blue line), aortic annulus diameter (right, green line), sinus of Valsalva diameter (right, red line), ST-segment junction diameter (right, yellow line), and ascending aorta diameter (right, white line). Abbreviations as in Figure 2.

**Figure 5:**
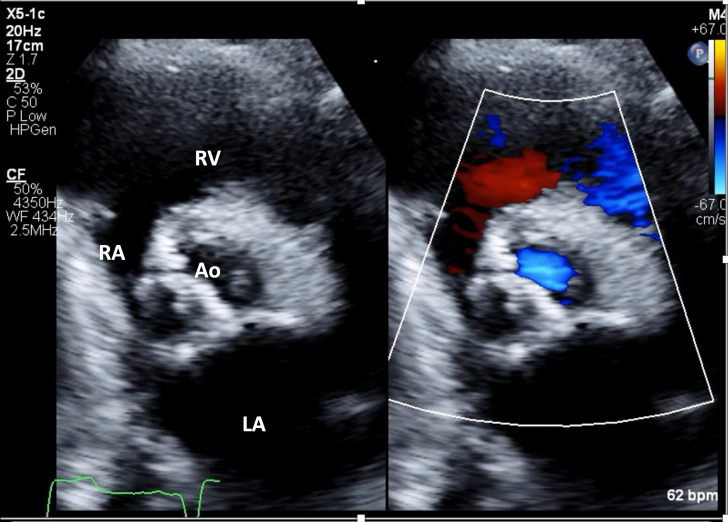
Short-Axis Evaluation of the Aortic Valve Parasternal short-axis view of the aortic valve used to interrogate valve morphology (identify bicuspid, tricuspid, etc) Note the location of calcium or presence of raphe. Abbreviations as in Figure 2.

**Figure 6:**
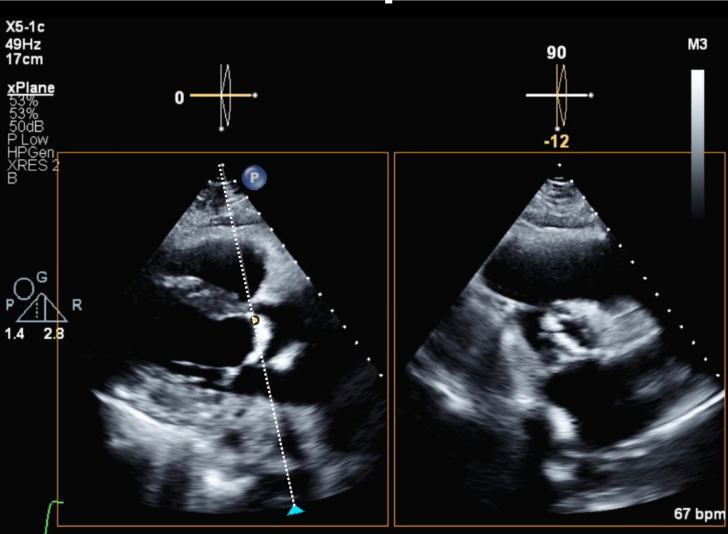
Planimetry of the Aortic Valve Area Direct planimetry of the aortic valve area can be performed using biplane where the cursor is placed at the leaflet tips. However, if not perfectly aligned at the leaflet tips, this may lead to error in direct planimetry measurement.

**Figure 7:**
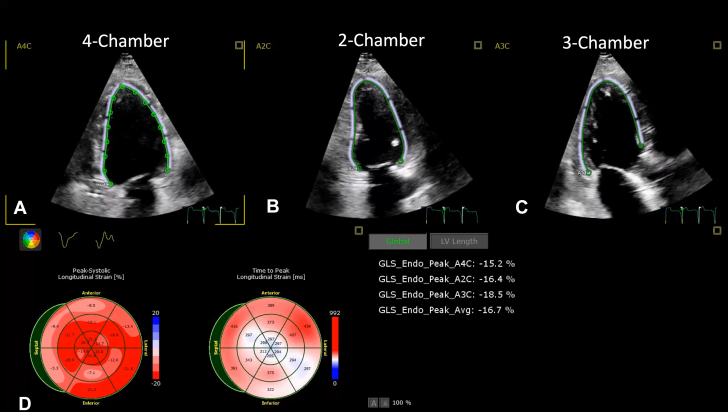
Left Ventricular Global Longitudinal Strain Left ventricular strain assessment using a 4-chamber view (A), 2-chamber view (B), and 3-chamber view (C). These images are analyzed and segmental longitudinal strain is shown in target fashion (D).

**Figure 8:**
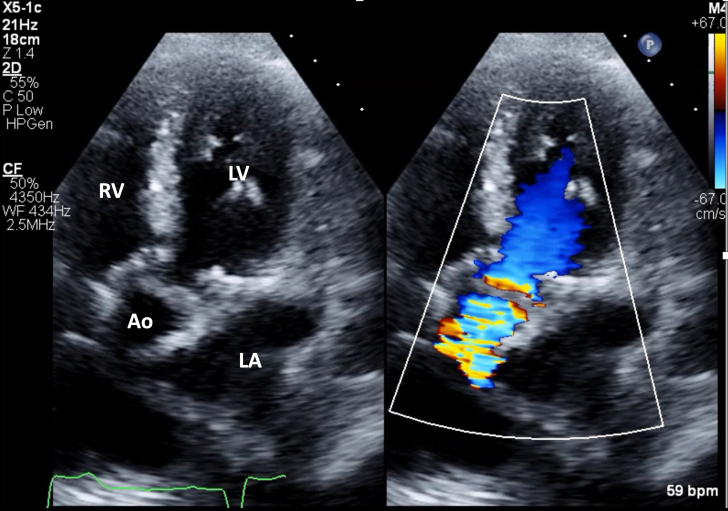
Apical 5-Chamber View Evaluation for Aortic Stenosis With and Without Color Doppler Apical 5-chamber view with color Doppler of the aortic valve to evaluate for the stenotic mosaic color flow pattern caused by an outflow obstruction and also for aortic regurgitation. Abbreviations as in Figure 2.

**Figure 9:**
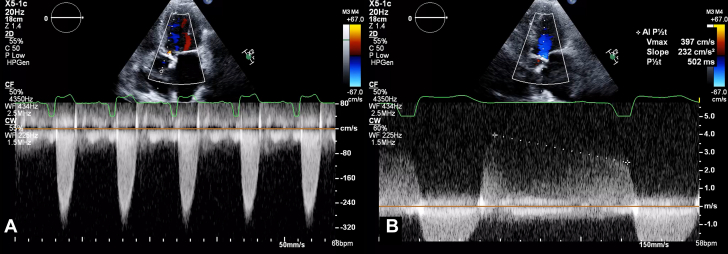
Apical 5-Chamber View Continuous- Wave Doppler Assessment Apical 5 chamber: align CW Doppler through the aortic valve to obtain aortic valve peak velocity (A) and gradients and Doppler assessment of aortic regurgitation (B).

**Figure 10:**
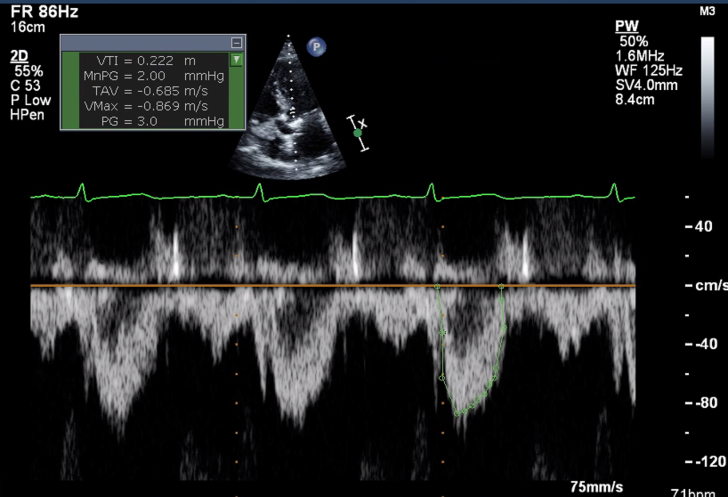
Apical 5-Chamber View Pulse Wave Doppler Assessment Apical 5 chamber: align PW sample volume in the left ventricular outflow tract avoiding the area of flow convergence.

**Figure 11:**
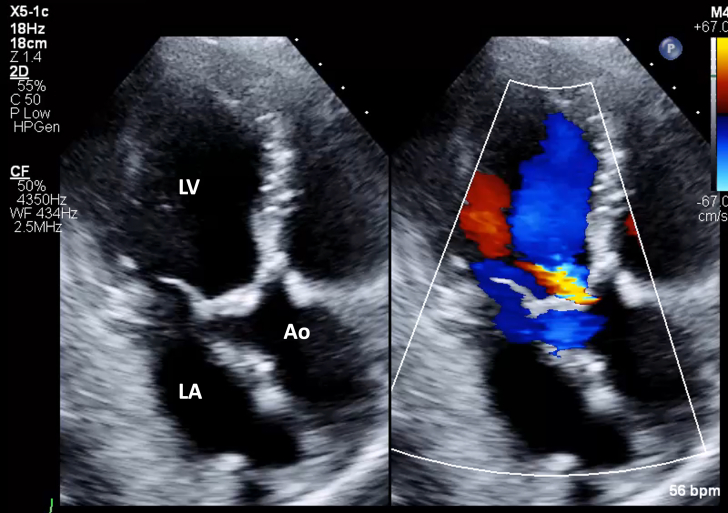
Apical 3-Chamber View Evaluation for Aortic Stenosis With and Without Color Doppler Apical 3 chamber: color Doppler on aortic valve to evaluate for aortic regurgitation and a stenotic mosaic color flow pattern caused by an outflow obstruction. Abbreviations as in Figure 2.

**Figure 12:**
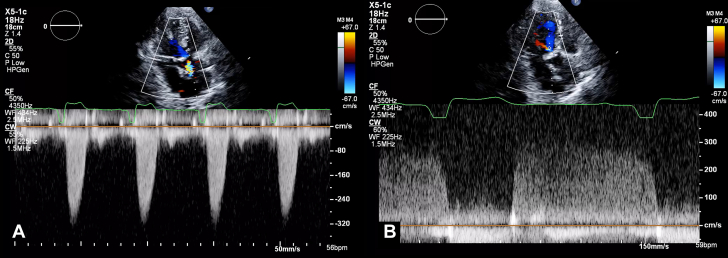
Apical 3-Chamber View Continuous -Wave Doppler Assessment Apical 3 chamber: align CW Doppler through the aortic valve to obtain aortic valve peak velocity, gradients, and Doppler assessment of aortic regurgitation.

**Figure 13:**
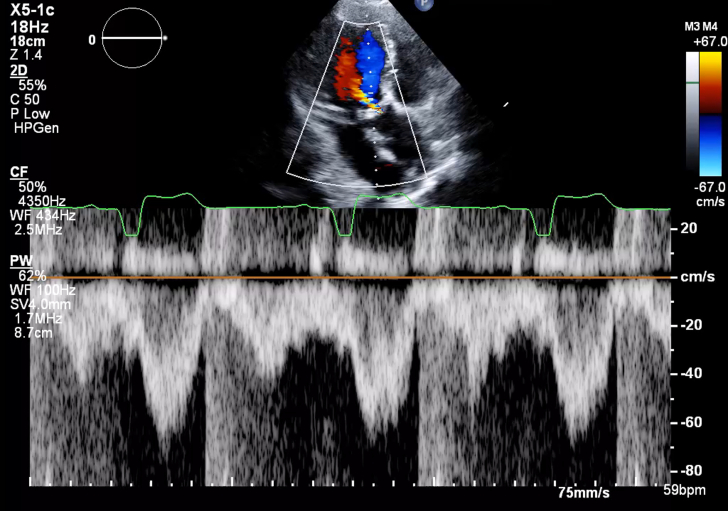
Apical 5-Chamber View Pulse Wave Doppler Assessment Apical 3 chamber: align PW sample volume in the left ventricular outflow tract avoiding the area of flow convergence.

**Figure 14:**
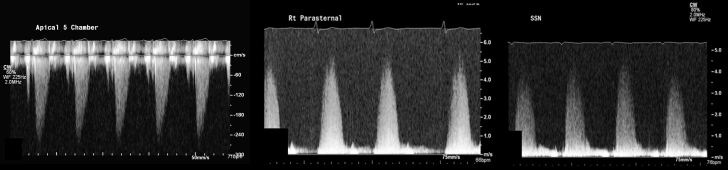
PEDOFF Probe Evaluation for Aortic Stenosis PEDOFF (nonimaging transducer) gradients obtained from the apical 5-chamber view, suprasternal notch, and right parasternal positions.

**Figure 15:**
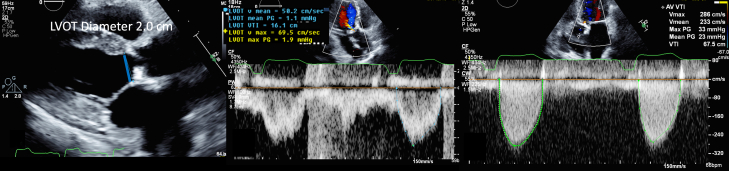
Calculated Aortic Valve Area by the Continuity Equation Aortic valve area is calculated by the continuity equation using the left ventricular outflow tract (LVOT) diameter from the parasternal long-axis view, the LVOT velocity, and aortic valve velocity.

**Figure 16:**
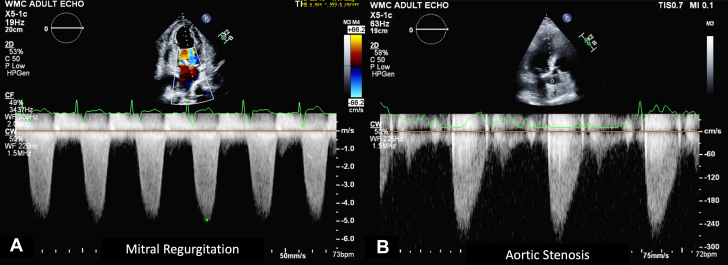
Comparing Mitral Regurgitation and Aortic Stenosis Doppler Profiles (A) Mitral regurgitation Doppler showing rounded jet contour that occurs during both the isovolumic contraction time and isovolemic relaxation time periods of the cardiac cycle. (B) Compared with aortic stenosis, Doppler that shows a rounded but slightly peaked velocity that occurs in systole after isovolemic contraction time but before isovolemic relaxation time.
2. Be sure to rule out other etiologies for outflow obstruction including both subvalvular and supravalvular stenosis. A subaortic membrane is a thin membrane that may be discrete or tunneled in the LVOT. When zooming in on the LVOT, look for a membrane and check for flow acceleration with color Doppler that occurs in the LVOT (below the AV). It is not uncommon to see associated aortic regurgitation in these patients. In patients with subvalvular narrowing from hypertrophic cardiomyopathy, the thickened ventricular septum narrows the LVOT. Systolic anterior motion of the anterior mitral valve leaflet may also be present leading to LVOT turbulence by color Doppler in the location of the narrowing and septal annular motion. The CW Doppler has a late peaking dagger-shaped appearance (Figure 17). PW cursor placement is extremely important in these patients with careful adjustment to avoid flow acceleration (Doppler aliasing) from the subvalvular obstruction.

**Figure 17:**
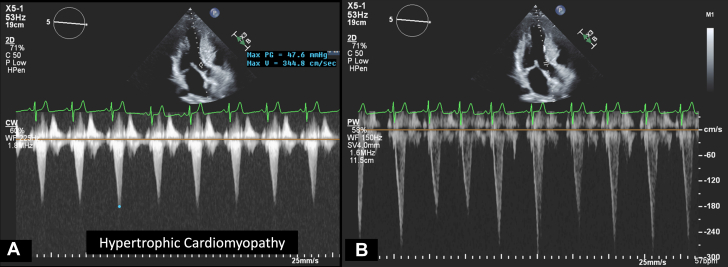
Doppler Profiles of a Patient With Hypertrophic Cardiomyopathy Continuous-wave Doppler of a patient with hypertrophic cardiomyopathy showing late peaking dagger-shaped CW Doppler waveform (A) and PW Doppler waveform showing increased velocity with aliasing (B).
3. Poor alignment of your Doppler cursor will lead to underestimation or overestimation of gradients. Improper placement of PW sample volume will overestimate (if too close to AV) or underestimate (if too far from AV) the left ventricular stroke volume, leading to inaccurate AV area measurement using the continuity equation. When positioning the PW Doppler sample volume in the LVOT, if spectral broadening artifact is occurring, reposition the sample volume away from the flow convergence. Alignment of the CW Doppler cursor as parallel to the direction of blood flow will maximize the AV velocity and gradients. In patients with heavily calcified AV, bicuspid AV, or tortious aortas, alignment of the CW cursor may be challenging. Using color Doppler can help identify the direction of blood flow and then best align the CW Doppler cursor. It is important to attempt imaging in all recommended views using both imaging and Pedoff (nonimaging) probes.
4. Ultrasound enhancing agents can be used to obtain gradients across the AV but require careful gain adjustments to avoid overestimation of the Doppler profile (Figure 18).

**Figure 18:**
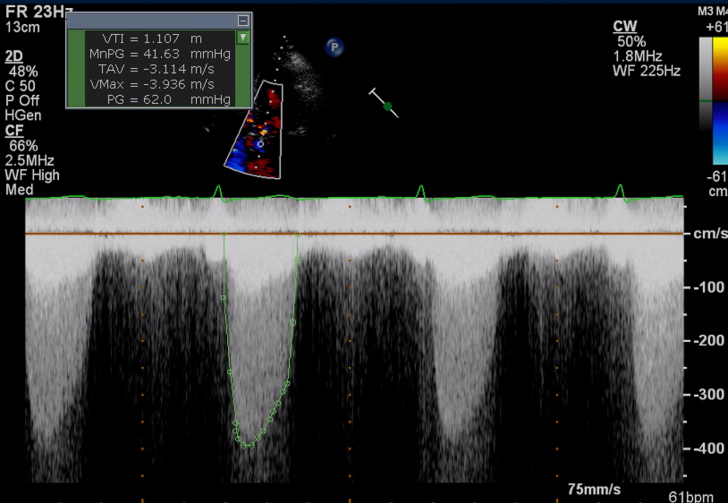
Aortic Velocities Using Ultrasound Enhancing Agents CW Doppler evaluation of a patient with aortic stenosis. The gain settings were reduced to be ensure that gradients are not overestimated.
5. Recognize LF-LG AS. This occurs when left ventricular stroke volume is low, leading to peak AV velocity or AV gradients that may not fulfill all the criteria for high gradient severe AS. For patients with severe LF-LG AS, the calculated AV area is <1.0 cm^2^, indexed stroke volume is <35 mL/m^2^, and dimensionless index is <0.25. Accurate measurement of the calculated stroke volume in these patients is vital with caution not to overestimate or underestimate the LVOT diameter or the LVOT PW velocities. Dobutamine stress echocardiogram may be ordered to confirm the diagnosis of severe LF-LG AS in certain patients.
6. Limitations of the continuity equation include the assumption that the LVOT area is perfectly round in its shape. In settings where the LVOT is elliptical in shape, the measured LVOT diameter in the PSLX view may underestimate the true LVOT area using the continuity equation. In situations where the LVOT is elliptical or eccentric in shape, using a 3-dimensional volume set to measure a direct planimetry area of the LVOT may be a reasonable work around.

## Conclusions

Echocardiography is a cost-effective and noninvasive tool providers can use in the early detection of AS. When an echocardiogram is performed correctly, the accuracy of the measurements and overall assessments of AS can lead to an earlier identification of disease state, early intervention, and overall, increased chance of patient survival.

## Funding Support and Author Disclosures

Dr Poniros has received speaker honoraria from Lantheus. Dr Tang has received speaker honoraria and served as a physician proctor, consultant, advisory board member, TAVR publications committee member, RESTORE study steering committee member, APOLLO trial screening committee member, and IMPACT MR steering committee member for Medtronic; has received speaker honoraria and served as a physician proctor, consultant, advisory board member, and TRILUMINATE trial anatomic eligibility and publications committee member for Abbott Structural Heart; has served as an advisory board member for Boston Scientific, a consultant and physician screening committee member for Shockwave Medical, a consultant for Philips and Edwards Lifesciences, Peija Medical, and Shenqi Medical Technology; and has received speaker honoraria from Siemens Healthineers. Dr Safi has received speaker honoraria for Abbott Structural Heart and served on advisory board for Triclip; and has received speaker honoraria from Medtronic. Dr Nevin has reported that she has no relationships relevant to the contents of this paper to disclose.

## Appendix

Video 1 Parasternal Long-Axis View of the Left Ventricle

Video 2 Parasternal Long-Axis View Using Color Doppler With Color Compare to Evaluate the Aortic Valve for Regurgitation and the Mosaic Color Flow Pattern Seen With Aortic Stenosis

Video 3 Parasternal Long-Axis View Using Color Doppler With Color Compare to Evaluate the Aortic Valve Enface

Video 4 Global Longitudinal Strain of the Left Ventricle Obtained Using the 4-Chamber, 2-Chamber, and 3-Chamber Views

Video 5 Apical 5-Chamber View Using Color Doppler With Color Compare of the Aortic Valve to Evaluate for the Stenotic Mosaic Color Flow Pattern Caused by an Outflow Obstruction and Also for Aortic Regurgitation

Video 6 Apical 3-Chamber View Using Color Doppler With Color Compare to Evaluate the Aortic Valve to Evaluate for Obstructive Mosaic Color Flow Patterns and to Also Evaluate for Aortic Regurgitation
